# Artificial Intelligence’s Use in the Diagnosis of Mouth Ulcers: A Systematic Review

**DOI:** 10.7759/cureus.45187

**Published:** 2023-09-13

**Authors:** Anushree Tiwari, Neha Gupta, Deepika Singla, Jnana Ranjan Swain, Ruchi Gupta, Dhaval Mehta, Santosh Kumar

**Affiliations:** 1 Clinical Quality and Value, American Academy of Orthopaedic Surgeons, Rosemont, USA; 2 Department of Oral Pathology, Microbiology & Forensic Odontology, Dental College, Rajendra Institute of Medical Sciences, Ranchi, IND; 3 Department of Conservative Dentistry & Endodontics, Desh Bhagat Dental College & Hospital, Malout, IND; 4 Department of Pedodontics and Preventive Dentistry, Institute of Dental Sciences, Siksha ’O’ Anusandhan, Bhubaneswar, IND; 5 Department of Prosthodontics, Rungta College of Dental Sciences and Research, Bhilai, IND; 6 Department of Oral Medicine and Radiology, Narsinbhai Patel Dental College and Hospital, Sankalchand Patel University, Visnagar, IND; 7 Department of Periodontology and Implantology, Karnavati School of Dentistry, Karnavati University, Gandhinagar, IND

**Keywords:** oral medicine, systematic, review, dentistry, oral ulcers, artificial intelligence

## Abstract

Artificial intelligence (AI) has been cited as being helpful in the diagnosis of diseases, the prediction of prognoses, and the development of patient-specific therapeutic strategies. AI can help dentists, in particular, when they need to make important judgments quickly. It can eliminate human mistakes in making decisions, resulting in superior and consistent medical treatment while lowering the workload on dentists. The existing studies relevant to the study and application of AI in the diagnosis of various forms of mouth ulcers are reviewed in this work. The Preferred Reporting Items for Systematic Reviews and Meta-Analyses (PRISMA) standards were followed in the preparation of the review. There were no rule violations, with the significant exception of the use of a better search method that led to more accurate findings. Using search terms mainly such as AI, oral health, oral ulcers, oral herpes simplex, oral lichen planus, pemphigus vulgaris, recurrent aphthous ulcer (RAU), oral cancer, premalignant and malignant disorders, etc., a comprehensive search was carried out in the reliable sources of literature, namely PubMed, Scopus, Embase, Web of Science, Ovid, Global Health, and PsycINFO. For all papers, exhaustive searches were done using inclusion criteria as well as exclusion criteria between June 28, 2018, and June 28, 2023. An AI framework for the automatic categorization of oral ulcers from oral clinical photographs was developed by the authors, and it performed satisfactorily. The newly designed AI model works better than the current convolutional neural network image categorization techniques and shows a fair level of precision in the classification of oral ulcers. However, despite being useful for identifying oral ulcers, the suggested technique needs a broader set of data for validation and training purposes before being used in clinical settings. Automated OCSCC identification using a deep learning-based technique is a quick, harmless, affordable, and practical approach to evaluating the effectiveness of cancer treatment. The categorization and identification of RAU lesions through the use of non-intrusive oral pictures using the previously developed ResNet50 and YOLOV algorithms demonstrated better accuracy as well as adequate potential for the future, which could be helpful in clinical practice. Moreover, the most reliable projections for the likelihood of the presence or absence of RAU were made by the optimized neural network. The authors also discovered variables associated with RAU that might be used as input information to build artificial neural networks that anticipate RAU.

## Introduction and background

Despite having distinct meanings, the terms artificial intelligence (AI) and machine learning (ML) are frequently used synonymously in studies. The term “artificial intelligence” was first used by John McCarthy, known as the pioneer of AI, to refer to computers that have the capacity to function in a way that would be regarded as intelligent, although devoid of any human involvement [1]. By taking into account the data input, these devices can solve issues. The earliest AI program was called Logic Theorist, created in 1955 by Allen Newell along with Herbert Simon [2,3]. AI has an important subset called ML [4], which was first used by Simon Cowell in 1959 [5]. Using tools such as artificial neural networks (ANN), ML makes predictions according to the information that is provided to it. Interrelated artificial nerves in these networks, which resemble the structure of the brain of a human being, take in and interpret data inputs.

Deep learning (DL), often known as a convolutional neural network (CNN), is a method for ML that Hinton et al. [3] developed in 2006. It computes data using multilayer ANNs. Algorithms based on DL are able to identify trends in the information and thus enhance the result. The evolution of backpropagation technology in 1969 [6-8] led to the use of DL models in various sectors, indicating a significant turning point in AI development over time. The use of AI technologies in the dentistry domain is rapidly evolving. Currently, mouth cancer is a significant public health issue [7-11]. Its diagnosis is frequently made at a late stage, making it quite challenging to manage the condition, and as a consequence, the fatality rate is high. Despite the various research initiatives taken to enhance the current therapy options, this has remained the same in the past few years [1,12]. Premalignant lesions (possibly indicative of a malignant illness) often observed in the oral cavity can be used to diagnose this form of cancer in a substantial percentage of cases [1,13-15].

Owing to inadequate knowledge about the medical conditions that cause mouth ulcers and their comparable looks, the identification and management of oral ulcers are frequently difficult. Even though persistent trauma is a common cause of oral ulcers, some of them may also be symptoms of a systemic disorder, such as gastrointestinal malfunction, cancer, immunologic abnormalities, or cutaneous diseases. Clinicians who treat individuals with oral mucosal diseases give high priority to making an accurate, conclusive diagnosis. Most of these illnesses are long-lasting, and patients experience symptoms, including desquamation; however, some may be contagious. It is necessary to comprehend the immunopathologic character of the lesion to administer effective treatment [16,17].

AI has been cited as being helpful in the diagnosis of diseases, the prediction of prognoses, and the development of patient-specific therapeutic strategies [17]. AI can help dentists, in particular, when they need to make important judgments quickly. It can eliminate human mistakes in making decisions, resulting in superior and consistent medical treatment while lowering the workload on dentists. However, the existing studies on the role of AI in oral ulcers do not follow an organized pattern. Therefore, the existing literature relevant to the study and application of AI in the diagnosis of various forms of mouth ulcers has been reviewed comprehensively in this work.

## Review

Search strategy

**Figure 1:**
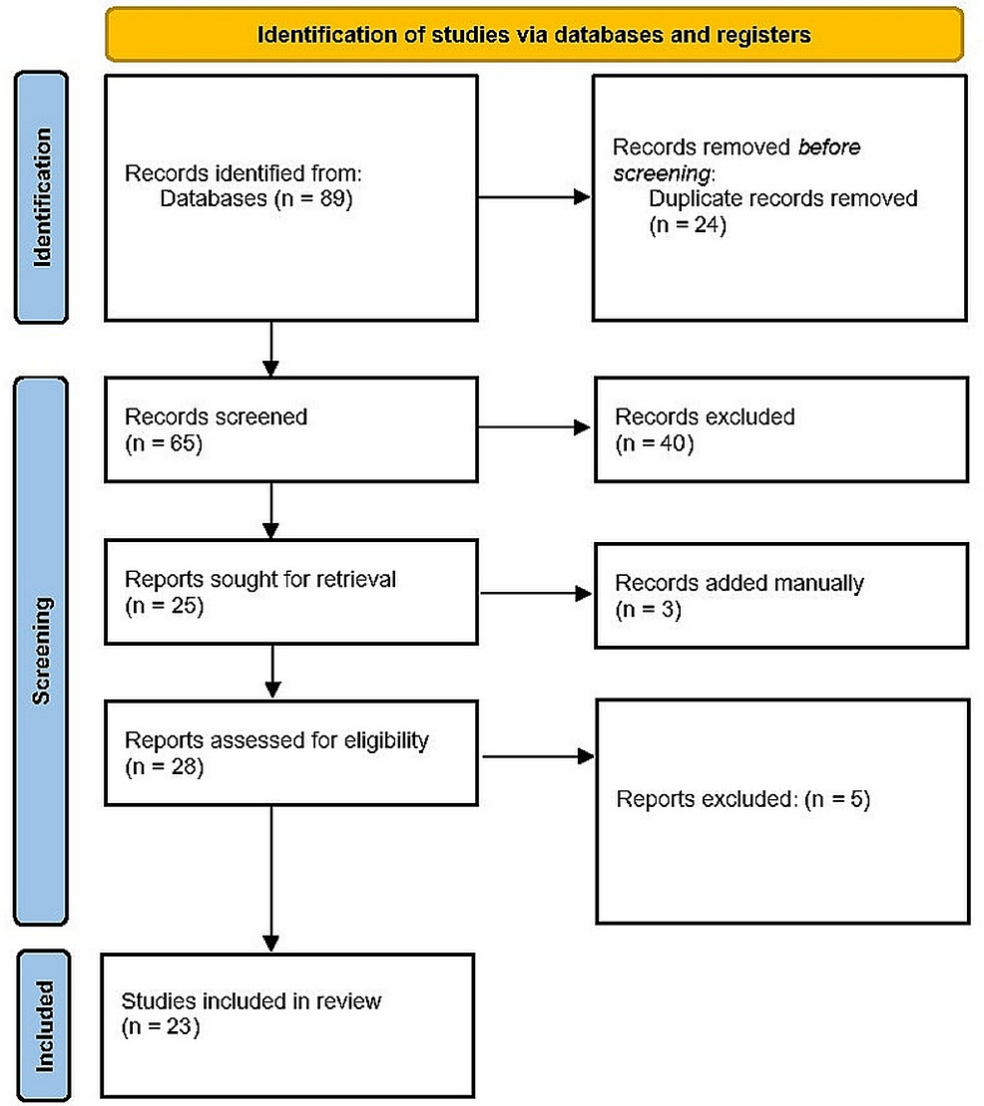
PRISMA flowchart showing the systematic selection of articles

The Preferred Reporting Items for Systematic Reviews and Meta-Analyses (PRISMA) standards were followed in the preparation of the review. No rules were violated, with the significant exception of the use of a better search method that led to more accurate findings (Figure 1). Using search terms such as AI (ML and DL), oral health, oral ulcers, oral herpes simplex, oral lichen planus, pemphigus vulgaris, recurrent apthous ulcers, oral cancer, premalignant and malignant disorders, etc., a comprehensive search was conducted in reliable sources of literature, including PubMed, Scopus, Embase, Web of Science, Ovid, Global Health, and PsycINFO. For all papers, exhaustive searches were done using inclusion criteria as well as exclusion criteria between June 28, 2018, and June 28, 2023.

Reviewers were trained in the two phases of carrying out eligibility testing for publications before being assigned to screening activities: full-text assessment and assessment of just the abstracts. Rayyan software was used to run the test using an abstract-only approach. The three investigators (XX, HH, and JJ) independently examined 33.33 percent of the overall search findings twice, while a single observer (AB) read through all of the results. The committee of reviewers convened to settle their disagreements after reading the abstracts in order to compile the final set of manuscripts that needed to be reviewed in full. Covidence software was used to perform a full-text review. The full-text papers were evaluated by two objective raters, WW and YY, in light of the standards. Investigators who were unsure if a particular approach had been used in a manuscript approached the authors of the concerned manuscript and requested more details. The final selection of publications that were decided to be taken into review was agreed upon by the panel’s members and evaluators from the scientific committee.

Inclusion criteria and exclusion criteria

The manuscripts relevant to AI, such as original research, literature reviews, scientific communications, systematic reviews, letters to editors, and many other preprints covering the usage of AI in the following categories, were included: (1) the diagnosis of oral ulcers; (2) the diagnosis of pemphigus vulgaris; (3) oral cancer and oral precancer; and (4) the diagnosis of oral herpes simplex infection, oral lichen planus, oral submucous fibrosis, and recurrent aphthous stomatitis. The following literature material was listed for exclusion: (1) written materials not in English; (2) documents pertaining to topics not included in the inclusion criteria; and (3) publications found in newspapers, periodicals, blogs, and other nonacademic sources.

Article selection

Eighty-nine papers were found using the search criteria. Twenty-four publications were excluded because they were duplicates or similar. Sixty-five different articles were first chosen. Following an examination of the titles and abstracts, 40 publications were removed. For 25 articles, full text management was done. The extra two papers were manually retrieved from references. There were 37 articles with full texts that could be read. Fifteen subpar articles were eliminated from the final evaluation. Finally, for this comprehensive assessment, 23 articles were chosen (Figure 1, Table 1).

**Table 1:** Important features of studies included in the systematic review

Author with year	Objective	Modality	Results	Conclusion
Gomes RFT et al., 2023 [4]	To execute the training as well as the validation of a CNN-based model that automatically categorizes six clinically representative types of oral ulcers photos.	The CNN model InceptionV3	The diagnosis of oral ulcers produced significant results utilizing an InceptionV3-based framework. The authors achieved over seventy-one percent accurate predictions in each of the six lesion categories after hyperparameter optimization. In the collection of data, the categorization had an average reliability of 95.09%.	An AI framework for the automatic categorization of oral ulcers from oral clinical photographs was developed, according to the authors, and it performed satisfactorily.
Guo J. et al., 2022 [5]	To suggest a modified residual network algorithm for instantaneous oral ulcer picture classification.	RN (Residual Network framework)	The findings from the experiment demonstrate that when mouth ulcers are identified and classified in real time, the method developed by the authors surpasses those traditional classification AI networks.	The authors' method works better than the current CNN image categorization techniques and has a fair level of precision in classification. Despite being useful for identifying oral ulcers, the suggested technique needs a broader set of data for validation and training purposes before being used in clinical settings.
Fu Q et al., 2020 [6]	To create a deep learning method that can quickly, non-invasively, cheaply, and simply detect people who have ulceropriliferative growth in the oral cavity corresponding to OSCC from clinical photographs.	DL CNN	Additionally, the approach greatly outperformed both ordinary medical students and ordinary non-medical students in terms of performance.	Automated OCSCC identification using a DL-based technique is a quick, harmless, affordable, and practical approach that has the features to be used as a clinical tool for quick evaluation, earlier identification, and evaluating the effectiveness of cancer treatment.
Zhou M et al., 2023 [7]	To assess CNNs for computerized categorization and identification of RAU, frequently occurring disorders of oral mucosa, and healthy oral mucosa in clinical images.	CNN models ResNet50 model YOLOV5 model	The Pretrained ResNet50 network performed exceptionally well in the process of categorizing, with an accuracy of 92.86 percent. The YOLOV5 model that had been developed had the greatest results, with an accuracy of 98.70 percent.	The categorization and identification of RAU lesions through the use of non-intrusive oral pictures using the previously developed ResNet50 and YOLOV5 algorithms were demonstrated to have better accuracy as well as adequate potential, which may be helpful in clinical practice.
Dar-Odeh NS et al., 2010 [8]	To build and improve an artificial neural network that can foretell the incidence of RAU using a collection of suitable input data.	ANN	The best reliable projections for the likelihood of the presence or absence of RAU were made by the optimized neural network.	The authors discovered variables associated with RAU that might be used as input information to build ANNs that anticipate RAU.
Speight PM et al., 1995 [9]	To assess a neural network's capacity to anticipate the possibility that a person would have a cancerous or potentially cancerous mouth lesion according to knowledge about their risk factors.	Neural network	In general, the dentists' accuracy and precision were 0.74 and 0.99, vs. 0.80 and 0.77 for the artificial neural network.	The aforementioned neural network could potentially be useful for recognizing people with an elevated likelihood of cancer of the oral cavity or precancer of the oral cavity to undergo additional clinical assessment or health awareness training in light of the potential expenses associated with running a screening strategy.
van Staveren HJ et al., 2000 [10]	An alternate approach to categorization for the autofluorescence spectroscopy of oral eryhtroplakia, which may indicate the degree of cellular dysplasia, was assessed to see how well it performed.	Pre-scaled spectra of artificial neural networks.	A neural network could identify between tissue and ulcers with significant accuracy.	The performance of categorizing whether erythroplakia is normal through AI was acceptable.
Wang CY et al., 2003 [11]	This study investigated the likelihood of separating human mouth precancerous as well as cancerous lesions from healthy or normal mouth tissue employing an ANN approach.	PLS-ANN algorithm	The PLS-ANN classification system had an average sensitivity of 81 percent, an accuracy of 96%, and a favorable prognostic value of 88% for separating precancerous tissues and cancerous tissues from normal tissues.	For in vivo identification of OSF in addition to oral precancerous and cancerous lesions, the PLS-ANN classification technique is helpful.
Paul RR et al., 2005 [12]	To provide a unique oral submucous fibrosis grade identification approach that utilizes artificial neural networks.	Artificial neural network	After receiving the photograph as an input, the network that had been trained was capable of classifying healthy stages and oral precancer conditions.	The outcomes of this technique's testing were encouraging, and they point to the possibility of using it for staging, recognizing OSF, and extending it to different settings with more refinement.
Nayak GS et al 2006 [13]	On a comparable set of spectrum data, spectral evaluation and categorization for differentiating between malignant, premalignant, and normal situations were carried out employing principal component analysis (PCA) and ANN individually.	PCA ANN	Both categorization algorithms' specificity as well as sensitivity were assessed. For the data set under consideration, they were 100 percent and 92.9 percent in the context of PCA and 100 and 96.5 percent in the scenario of ANN, respectively.	ANN may help distinguish between cancerous conditions and precancerous conditions.
Kim JS et al., 2022 [14]	On the basis of pictures of the oral mucosa, the effectiveness of AI-related identification of oral malignancy conditions was assessed.	AI, ML, ANN	AI-assisted computerized recognition of oral malignancy conditions could serve as a quick, harmless diagnostic aid that may give instantaneous conclusions on the evaluation for the diagnosis of cancer of the mouth.	A practical approach for the timely identification of pathogenic lesions may be created using this AI technique.
Duran-Sierra E et al., 2021 [15]	This research explored the possibility of using maFLIM-derived autofluorescence indicators in ML frameworks to distinguish between normal oral tissue and malignant oral tissue.	ML, biomarkers of maFLIM-derived autofluorescence	In the present investigation, autofluorescence indicators that are frequently employed in ML frameworks can be used to identify malignant tissue of the oral cavity.	Widefield maFLIM endoscopy, in association with AI, has the ability to automatically recognize cancerous conditions of the oral cavity.
Noyan MA et al., 2020 [16]	To create the TzanckNet DL framework, which can recognize cells within the Tzanck smear.	TzanckNet model DL	TzanckNet generated 2154 estimations for 359 pictures and six different kinds of cells. Precision was 97.3 percent, responsiveness was 83.7 percent, and correctness was 94.3 percent.	The findings demonstrate that TzanckNet possesses the capability to reduce the expertise requirement for using this procedure, thereby increasing the number of its users and enhancing the overall health of patients.
Cai D. et al., 2021 [17]	To build a DL-based screening tool for autoimmune blistering diseases using a unique AI technique.	CNN Transfer learning	The most recent version maintains a level of diagnostic precision for more prevalent skin malignancies that is almost on the same level as the accuracy of dermatologists in diagnosing them.	This method has the ability to aid in the medical diagnosis of AIBDs, which is attributed to the prediction framework's effectiveness despite having inadequate training information.
Dubey S. et al., 2023 [18]	To conduct a comparison study and recommend an AI technique for PV diagnosis in the early phases of skin degeneration.	CNN	A comparison of the results using CNN was carried out with 78.7 percent precision.	The effectiveness of the suggested AI framework for detecting PV was established.
Yu K. et al. 2020 [19]	To review research on ML's use in psoriasis therapy and assessment and to talk about the prospects and obstacles for new developments.	Machine learning	ML has a lot of ability to help in the diagnosis and treatment of psoriasis. The interpretation of medical photographs, the foretelling of consequences, and the development of treatments are currently hot areas in ML studies regarding psoriasis.	Dermatologists would benefit from knowing more about ML and how it might enhance evaluations and decisions, with the goal of allowing patients to profit the most from ML breakthroughs.
Achararit et al., 2020 [20]	To utilize a CNN framework of AI to identify the lichen planus of the oral cavity (OLP) in histopathologically confirmed clinical OLP.	CNN model and Xception framework	All of the chosen CNN models were capable of using images to identify OLP abnormalities. In regard to both general precision and F1 rating, the model built using Xception performed much better than the other approaches.	As demonstrated by their experiment, CNN-based algorithms are capable of achieving an estimated precision of 82 percent to eighty-eight percent. Reliability and F1-score were best achieved with the Xception algorithm.
He X et al., 2020 [21]	To create and assess an AI algorithm for the diagnosis of bullous pemphigoid (BP) and PV.	ML, AI	The AI-based diagnostic structure, which was built on clinical imaging and clinical information, identified BP and PV at a level comparable to dental expert standards.	Dermatologists in rural locations may find this AI-based diagnostic paradigm useful in the early detection of each of these disorders.
Surodina S. et al. [22]	By discovering indicators of HSV infection along with choosing just a handful of pertinent questions that could be asked with new register members to ascertain their probability of HSV risk for infection, this research sought to enhance data collection for an everyday life HSV database.	Random forest algorithm	The model chose fewer items that accurately predicted the likelihood of being infected with HSV with significant high ratings and accurately remembered the danger for HSV-1 as well as HSV-2 data sets.	In an everyday evidence database, this ML system can be utilized to gather pertinent lifestyle information and determine every individual's degree of likelihood of contracting HSV infection.
Natarajan R. et al. [23]	To use and investigate DL techniques based on AI for herpes simplex diagnosis.	Deep learning algorithms, DenseNet, ResNet	DenseNet outperformed ResNet as well as Inception in the particular assignment with a greater level of precision.	The DL approach can be a helpful tool for diagnosing HSV, particularly in primary care settings without access to the necessary laboratory equipment or skilled personnel.
Nowell WB, 2021 [24]	To elaborate on the procedure for creating and putting into use an AI-based approach to lay the framework for creating a patient database for those who are in danger of contracting or already have HSV.	DL	The number of inquiries required to obtain a high degree of precision when forecasting HSV infection was optimized by the researchers employing this technique.	The authors place their novel approach in the broader perspective of both the difficulties in developing a patient register for a stigmatized ailment and the chances to establish new registries by utilizing publicly accessible. data sets.
Idrees M. et al., 2021 [25]	To assess AI's contribution to the diagnosis of OLP.	ML, AI	The proposed machine-learning method was reliably capable of detecting OLP cases based on the number of inflammatory cells and the number of mononuclear cells.	Oral pathologists can more effectively diagnose OLP using aspects of its development thanks to AI, which has demonstrated encouraging results and offers a robust method.
Keser G. et al., 2023 [26]	To create a DL method to recognize OLP lesions from picture photos.	AI, Google Inception V3, Tensorflow, and DL	All experimental photos were correctly classified as represented by an AI framework, with an accuracy of one hundred percent.	The initial outcomes demonstrate that AI possesses the capability to address this important topic.

Characteristics of manuscripts

The manuscripts that were included covered various aspects of AI in different types of oral ulcers, such as oral lichen planus, pemphigus vulgaris, recurrent apthous ulcers, herpes simplex virus infection, psoriasis, oral submucous fibrosis, oral cancer, and precancerous ulcerative lesions. The AI models that were used were R, ML, DL, and RL. The objective of the study, the methodology used, the results obtained, and the conclusions drawn from each manuscript were collected.

AI in recurrent apthous ulceration

In 2023, Zhou et al. [7] conducted a study to assess CNNs in computerized categorization and identification of recurrent aphthous ulcer (RAU), frequently occurring disorders of the oral mucosa, and healthy oral mucosa through clinical images. The pretrained ResNet50 network performed exceptionally well in the process of categorization, providing an accuracy of 92.86 percent. The YOLOV5 model gave the best result, with an accuracy of 98.70 percent. The categorization and identification of RAU lesions through nonintrusive oral pictures using the previously developed ResNet50 and YOLOV5 algorithms demonstrated better accuracy as well as adequate potential for the future, which could be helpful in clinical practice.

Using a collection of suitable input data in 2010, Dar-Odeh et al. [8] conducted a study to build an optimized ANN that could give the most reliable projections for the likelihood of the presence or absence of RAU. The authors discovered variables associated with RAU that could be used as input information to build ANNs that anticipate RAU. Guo et al. [5] performed a study in 2022 to suggest a modified residual network algorithm for instantaneous oral ulcer picture classification. The findings from this experiment demonstrated that the method developed by the authors to identify and categorize mouth ulcers in actual time worked better than the current CNN image categorization techniques as it displayed a fair level of precision in classification, surpassing the traditional classification AI networks. However, despite being useful in identifying oral ulcers, the suggested technique needs a broader set of data for validation and training purposes before being used in clinical settings.

Gomes et al. [4] also conducted a study in 2023 to execute the training as well as the validation of a CNN-based model that automatically categorized six clinically representative types of oral lesion photos. Utilizing an inceptionV3-based framework, the diagnosis of oral ulcers produced significant results. The authors achieved over 71% accurate predictions in each of the six lesion categories after hyperparameter optimization. In addition, in the collection of data, the categorization showed an average reliability of 95.09%. An AI framework for the automatic categorization of oral ulcers from oral clinical photographs was developed, according to the authors, which performed satisfactorily.

AI in oral lichen planus

In 2020, Achararit et al. [20] conducted a study to identify the lichen planus of the oral cavity (OLP) in histopathologically confirmed clinical OLP by utilizing a CNN framework of AI. All of the chosen CNN models were capable of using images to identify OLP abnormalities. CNN model and Xception algorithm were modalities tested. In regard to both general precision and F1 rating, the model built using Xception performed much better than the other approaches. As demonstrated by their experiment, CNN-based algorithms were capable of achieving an estimated precision of 82%-88%. Reliability and F1-score were best achieved with the Xception algorithm.

In 2021, Idrees et al. [25] conducted a study to assess AI’s contribution to the diagnosis of OLP. The machine-learning method proposed by them was capable of reliably detecting OLP cases based on the number of inflammatory cells and the number of mononuclear cells. It was concluded that oral pathologists can more effectively diagnose OLP using aspects of its development owing to AI, which has demonstrated encouraging results and offers a robust method. Keser et al. [26] also carried out research in 2023 for creating a DL method to recognize OLP lesions from picture photos. All experimental photos were correctly classified by an AI framework, with an accuracy of 100%. The initial outcomes demonstrate that AI possesses the capability of addressing this important topic.

AI in pemphigus vulgaris, bullous pemphigoid, autoimmune blistering disease, psoriasis

In 2020, He et al. [21] carried out a study to create and assess an AI algorithm for the diagnosis of bullous pemphigoid (BP) and PV. The AI-based diagnostic structure, built on clinical imaging and information, identified BP and PV at a level comparable to dental expert standards. Dermatologists in rural locations may find this AI-based diagnostic paradigm useful in the early detection of both of these disorders. Similarly, Dubey et al. [18] performed a comparison study in 2023 and recommended an AI technique for PV diagnosis in the early phases of skin degeneration. A comparison of the results using CNN was carried out with 78.7 percent precision, thus establishing the AI framework’s effectiveness in detecting PV.

In 2021, Cai et al. [17] conducted a study to build a DL-based screening tool for the diagnosis of autoimmune blistering diseases (AIBD) using a unique AI technique. The most recent version maintains a level of diagnostic precision for more prevalent skin malignancies that is almost at the same level of accuracy as the diagnosis by a dermatologist. This method has the ability to aid in the medical diagnosis of AIBDs, which is attributed to the prediction framework’s effectiveness despite having inadequate training information.

Yu et al. [19] conducted a systematic review in 2020 on ML’s use in psoriasis therapy and assessment and the prospects and obstacles to its future development. ML has the ability to help in the diagnosis and treatment of psoriasis. The interpretation of medical photographs, the foretelling of consequences, and the development of treatments are currently hot areas in ML studies on psoriasis, and dermatologists would benefit from knowing more about ML and how it could enhance evaluations and decision-making. The final goal would be to allow patients to profit the most from ML breakthroughs.

AI in HSV infection

By discovering indicators of HSV infection along with choosing just a handful of pertinent questions that could be asked with newly registered members for ascertaining their probability of HSV risk for infection, the research by Surodina et al. [22] sought to enhance data collection for an everyday-life HSV database. This model chose fewer items to accurately predict the likelihood of being infected with HSV with significant high ratings while accurately considering the dangers of the HSV-1 and HSV-2 data sets. In the everyday evidence database, this ML system was utilized to gather pertinent lifestyle information and determine every individual’s degree of likelihood of contracting HSV infection.

In addition, Natarajan et al. [23] conducted a study to use and investigate DL techniques based on AI for the diagnosis of herpes simplex. AI models such as DL, DenseNet, and ResNet were evaluated. DenseNet outperformed ResNet as well as inception in the particular assignment regarding a greater level of precision. On the other hand, the DL approach was found to be a helpful tool for diagnosing HSV, particularly in primary care settings without access to the necessary laboratory equipment or skilled personnel.

In 2021, Nowell [24] conducted a study in which the number of inquiries required to obtain a high degree of precision in forecasting HSV infection was optimized. The author adopted this novel approach from the broader perspective of both difficulties in registering a patient for a stigmatized ailment and establishing new registries by utilizing publicly accessible data sets.

Noyan et al. [16] conducted a study to create the TzanckNet DL framework, which could recognize cells in a Tzanck smear. TzanckNet generated 2154 estimations for 359 pictures and six different kinds of cells. Precision was 97.3%, responsiveness was 83.7%, and correctness was 94.3%. The findings demonstrate that TzanckNet possesses the capability of reducing the expertise requirement for using this procedure, thereby increasing the number of its users and enhancing the overall health of patients.

AI in ulceroproliferative growth, oral cancer, precancer, OSMF

Fu et al. [6] conducted a study in 2020 to create a DL method that could quickly, noninvasively, cheaply, and simply detect people having ulceroproliferative growth in the oral cavity, corresponding to OSCC, from photographs. Additionally, the approach significantly outperformed both ordinary medical students and ordinary nonmedical students in terms of performance. Automated OCSCC identification using a DL-based technique is a quick, harmless, affordable, and practical approach that has the features of a clinical tool for quick evaluation, early identification, and evaluating the effectiveness of cancer treatment.

Similarly, in 1995, Speight et al. [9] performed a study on a neural network’s capacity to assess the possibility that a person could have a potentially cancerous mouth lesion, considering their risk factors. In general, the dentists’ accuracy and precision were 0.74 and 0.99, versus 0.80 and 0.77 for the ANN. The aforementioned neural network was useful in identifying people with a higher likelihood of cancer or precancer of the oral cavity for undergoing additional clinical assessment or health awareness training in light of the potential expenses associated with running a screening strategy. An alternate approach to categorization of oral eryhtroplakia by autofluorescence spectroscopy, which could indicate the degree of cellular dysplasia, was assessed by van Staveren et al. in 2000 [10]. The neural network could differentiate between pathological and healthy tissues with a degree of sensitivity of 86% and validity of 100%. Categorization of tissue, whether erythroplakia or non-erythroplakia tissue, through AI was acceptable.

Wang et al. [11] conducted a study in 2003 that investigated the likelihood of separating human mouth precancerous as well as cancerous lesions from healthy or normal mouth tissue employing an ANN approach. The PLS-ANN classification system displayed an average sensitivity of 81%, an accuracy of 96%, and a favorable prognostic value of 88% for separating precancerous tissues and cancerous tissues from normal tissues. For in vivo identification of OSF in addition to oral precancerous and cancerous lesions, the PLS-ANN classification technique was helpful.

Discussion

The application of AI technologies could have a substantial impact in the field of dentistry, especially in the current scenario that indicates mouth cancer is a serious public health concern [7,11]. Its late diagnosis makes treatment more difficult and leads to a higher fatality rate. Despite numerous research initiatives to improve the available therapeutic alternatives, the scenario has remained the same over the past few years [1,12]. However, in a significant number of cases, premalignant lesions (potentially malignant disorders) frequently observed in the oral cavity can be used for the diagnosis of this type of cancer [1,13-15].

Although oral ulcers could be mostly due to persistent trauma, some of them could also be signs of a more serious condition, such as gastrointestinal problems, cancer, immunologic issues, or skin illnesses. The precise and thorough diagnosis of patients with oral mucosal disorders is of utmost importance for clinicians to treat them. The bulk of these disorders are chronic, symptomatic, and desquamative; some are communicable, though. To provide appropriate treatment, it is essential to understand the nature of the lesion.

AI has been credited with aiding in disease diagnosis, prognosis prediction, and the creation of patient-specific therapy approaches [25]. Dentists, in particular, can benefit from AI when they need to swiftly make critical decisions. It can reduce the workload for dentists while removing human error from decision-making, leading to superior and consistent medical care. This paper reviews the existing literature pertinent to research on the use of AI in the diagnosis of various types of mouth ulcers.

The manuscripts included covered the various aspects of AI in different types of oral ulcers, which included oral lichen planus, pemphigus vulgaris, recurrent apthous ulcers, herpes simplex virus infection, psoriasis, oral submucous fibrosis, oral cancer, and precancerous ulcerative lesions. The AI models used were R, ML, DL, and RL. The objective of the study, the methodology used, the results obtained, and the conclusions drawn from each manuscript were gathered. Guo et al. [5] performed a study in 2022 to suggest a modified residual network algorithm for instantaneous oral ulcer picture classification. The findings from their experiment demonstrated that the authors’ method worked better in identifying and categorizing mouth ulcers than the current CNN image categorization techniques, as it displayed a fair level of precision. However, this needs a broader set of data for validation and training purposes before being used in clinical settings. Similarly, in 2023, Gomes et al. [4] conducted a study to execute the training and validation of a CNN-based model that automatically categorized six clinically representative types of oral lesion photos. The identification of oral ulcerative lesions produced the best results by utilizing an InceptionV3-based framework. The authors achieved over 71% accuracy in the predictions in each of the six lesion categories after hyperparameter optimization. In the collection of data, the categorization demonstrated an average reliability of 95.09%. An AI framework for the automatic categorization of oral ulcers from oral clinical photographs was developed by the authors and performed satisfactorily.

A few studies have investigated the role of AI in the diagnosis of RAU. In 2023, Zhou et al. [7] carried out a study to assess CNNs for computerized categorization and identification of RAU, frequently occurring disorders of oral mucosa, and healthy oral mucosa through clinical images. The pretrained ResNet50 network performed exceptionally well in categorization, with an accuracy of 92.86%, while the YOLOV5 model gave the best result, with an accuracy of 98.70%. Thus, the categorization and identification of RAU lesions through the use of nonintrusive oral pictures using the ResNet50 and YOLOV5 algorithms demonstrated better accuracy as well as adequate potential, which could be helpful in clinical practice. In addition, in 2010, Dar-Odeh et al. [8] built an optimized ANN that provided the best reliable projections for the likelihood of the presence or absence of RAU. Moreover, researchers have discovered variables associated with RAU that might be used as input information to build ANNs that anticipate RAU.

The terms “AI” and “ML”, despite having different meanings, are frequently used interchangeably in studies. John McCarthy, regarded as the father of AI, coined the term “artificial intelligence” to describe computers as having the ability to behave in a way that would be considered intelligent in the absence of human intervention [1]. These gadgets could resolve problems by considering the data input. ML is a significant subset of AI [4], which was coined by Simon Cowell in 1959 [5]. ML uses technologies such as ANNs to create predictions based on the data that is provided to it. These networks’ interconnected artificial nerves, which closely mirror the human brain’s structure, receive and process data inputs.

There has been some research related to the role of AI in the diagnosis of oral cancer ulcerative lesions. Fu et al. [6] conducted research in 2020 to create a DL method that could quickly, noninvasively, cheaply, and simply detect people having ulceroproliferative growth in the oral cavity corresponding to OSCC displayed in photographs. Additionally, the approach greatly outperformed both ordinary medical students and ordinary nonmedical students. Automated OCSCC identification using a DL-based technique is a quick, harmless, affordable, and practical approach that has the features of a clinical tool for quick assessment, early identification, and evaluation of the effectiveness of cancer treatment. In 2000, van Staveren et al. [10] provided an alternate approach to categorization of oral eryhtroplakia by autofluorescence spectroscopy, which indicated the degree of cellular dysplasia. The performance of categorizing erythroplakia or non-erythroplakia tissue through AI was acceptable. Wang et al. [11] conducted a study in 2003 to investigate the likelihood of separating human mouth precancerous as well as cancerous lesions from healthy or normal mouth tissue employing an ANN approach. The PLS-ANN classification system showed an average sensitivity of 81%, an accuracy of 96%, and a favorable prognostic value of 88% for separating precancerous tissues and cancerous tissues from normal tissues. For in vivo identification of OSF in addition to oral precancerous and cancerous lesions, the PLS-ANN classification technique is helpful.

DL-based algorithms might be able to spot trends in the data and improve the outcome. These systems were made possible by the development of back propagation technology in 1969 [8], indicating the turning point in the evolution of AI over time. Several authors have demonstrated the role of AI in the diagnosis of HSV [22-24]. By discovering indicators of HSV infection along with choosing just a handful of pertinent questions that could be asked with newly registered members to ascertain their probability of HSV risk for infection, Surodina et al.’s [22] research sought to enhance data collection for an everyday-life HSV database. The model chose fewer items to accurately predict the likelihood of being infected with HSV with significant high ratings while accurately considering the dangers of the HSV-1 and HSV-2 data sets. In an everyday evidence database, this ML system gathered pertinent lifestyle information and determined every individual’s degree of likelihood of contracting HSV infection. Natarajan et al. [23] conducted a study on AI-based DL techniques for the diagnosis of herpes simplex, including evaluating DL, DenseNet, and ResNet models of AI. DenseNet outperformed ResNet as well as Inception in the particular assignment with regards to a greater level of precision. The DL approach was found to be a helpful tool for diagnosing HSV, particularly in primary care settings without access to the necessary laboratory equipment or skilled personnel. Nowell [24] conducted a study in 2021 on the number of inquiries required to obtain a high degree of precision when forecasting HSV infection, which was optimized by the researchers employing this technique [25, 26]. The authors adopted this novel approach from the broader perspective of both difficulties in developing a patient register for a stigmatized ailment and establishing new registries by utilizing publicly accessible data sets. Noyan et al. [16] conducted a study in 2020 to create the TzanckNet DL framework, which could recognize cells in a Tzanck smear. TzanckNet generated 2154 estimations for 359 pictures and six different kinds of cells. The precision of this technique was 97.3%, responsiveness was 83.7%, and correctness was 94.3%, which demonstrate that it possesses the capability of reducing the expertise requirement for using this procedure, hence increasing the number of its users and enhancing the overall health of patients.

## Conclusions

In summary, an AI framework was developed by the authors for the automatic categorization of oral ulcers from oral clinical photographs, and it performed satisfactorily. The newly designed AI model worked better than the current CNN image categorization techniques and displayed a fair level of precision in the classification of oral ulcers. Despite being useful for identifying oral ulcers, the suggested technique needs a broader set of data for validation and training purposes before being used in clinical settings. Automated OCSCC identification using a DL-based technique is a quick, harmless, affordable, and practical approach for evaluating the effectiveness of cancer treatment. The categorization and identification of RAU lesions through the use of nonintrusive oral pictures using the previously developed ResNet50 and YOLOV5 algorithms demonstrated better accuracy as well as adequate potential for future use, which could be helpful in clinical practice. The best reliable projections for the likelihood of the presence or absence of RAU were made by the optimized neural network. In addition, the authors discovered variables associated with RAU that could be used as input information to build ANNs that anticipate RAU.

AI has the capability to enhance diagnostics by improving accuracy and enabling earlier disease detection. This could ultimately lead to improved patient outcomes and a reduction in invasive procedures. Additionally, AI can provide personalized treatment plans by analyzing large datasets and patient-specific information. Dentists may also benefit from streamlined workflows and reduced administrative tasks, allowing for more focused patient care. Furthermore, AI-powered tools, such as chatbots and virtual assistants, can enhance patient interactions and satisfaction. However, challenges must be considered, including data privacy, the need for continuous algorithm validation, ethical concerns surrounding decision responsibility and bias, ongoing education and training for dental professionals, and the cost and accessibility of AI technology. As AI continues to evolve in dentistry, addressing these challenges will be crucial to realizing its full potential.
